# Impact of Probiotics on the Glycemic Control of Pediatric and Adolescent Individuals with Type 1 Diabetes: A Systematic Review and Meta-Analysis

**DOI:** 10.3390/nu16162629

**Published:** 2024-08-09

**Authors:** Charikleia Stefanaki, Paraskevi Rozou, Vasiliki Efthymiou, Ioannis Xinias, George Mastorakos, Flora Bacopoulou, Maria Papagianni

**Affiliations:** 1 Department of Pharmacology, Medical School, National and Kapodistrian University of Athens, 11527 Athens, Greece; 2 Center for Adolescent Medicine and UNESCO Chair in Adolescent Health Care, First Department of Pediatrics, School of Medicine, National and Kapodistrian University of Athens, 11527 Athens, Greece; 3 Hygiene, Social & Preventive Medicine and Medical Statistics Laboratory, Medical School, Aristotle University of Thessaloniki, 54642 Thessaloniki, Greece; 4 Department of Pediatrics, General Hospital of Larisa, 38221 Larissa, Greece; 5 Third Pediatric Department, Hippokration Hospital, 54642 Thessaloniki, Greece; 6 Unit of Endocrinology, Diabetes Mellitus and Metabolism, Aretaieion Hospital, Athens Medical School, National and Kapodistrian University of Athens, 11528 Athens, Greece; 7 Endocrine Unit, 3rd Department of Pediatrics, Hippokration Hospital of Thessaloniki, Aristotle University of Thessaloniki, 54642 Thessaloniki, Greece

**Keywords:** T1D, probiotics, glycemic control, HbA1c, mean glucose concentrations, clinical pharmacology, children, adolescents, pediatrics

## Abstract

Aims: Human recombinant insulin is currently the only therapy for children and adolescents with type 1 diabetes (T1D), although not always efficient for the glycemic control of these individuals. The interrelation between the gut microbiome and the glycemic control of apparently healthy populations, as well as various populations with diabetes, is undeniable. Probiotics are biotherapeutics that deliver active components to various targets, primarily the gastrointestinal tract. This systematic review and meta-analysis examined the effect of the administration of probiotics on the glycemic control of pediatric and adolescent individuals with T1D. Materials and Methods: Randomized controlled trials employing the administration of probiotics in children and adolescents with T1D (with ≥10 individuals per treatment arm), written in English, providing parameters of glycemic control, such as mean glucose concentrations and glycosylated hemoglobin (HbA1c), were deemed eligible. Results: The search strategy resulted in six papers with contradictory findings. Ultimately, five studies of acceptable quality, comprising 388 children and adolescents with T1D, were included in the meta-analysis. Employing a random and fixed effects model revealed statistically significant negative effect sizes of probiotics on the glycemic control of those individuals, i.e., higher concentrations of glucose and HbA1c than controls. Conclusions: Children and adolescents with T1D who received probiotics demonstrated worse glycemic control than controls after the intervention. Adequately powered studies, with extended follow-up periods, along with monitoring of compliance and employing the proper strains, are required to unravel the mechanisms of action and the relative effects of probiotics, particularly concerning diabetes-related complications and metabolic outcomes.

## 1. Introduction

Type 1 diabetes (T1D) is a chronic autoimmune disease, combining insulin deficiency due to impaired pancreatic function and long-term micro- and macro-complications due to impaired glycemic control [1].

Administration of human recombinant insulin regimens, either by self-administered pens or by automated delivery systems [2,3], is currently the only therapy for pediatric and adolescent individuals with T1D, although not always efficient. While sodium-dependent glucose transporters (SGLT) inhibitors have been approved by the European Medicines Agency (EMA) and glucagon-like peptide 1 (GLP-1) agonists, along with metformin, amylin mimetics, dipeptidyl peptidase 4 (DPP4) inhibitors, or even humanized monoclonal antibodies to CD3 on T cells, such as teplizumab, have been used with success in adult individuals with T1D [4,5] and in newly diagnosed pediatric individuals with T1D, respectively [6,7,8], the on- and off-label armamentarium against T1D in pediatric and adolescent individuals is still very poor.

Despite the significant progress that has been achieved over the years in insulin administration and in glucose monitoring, there is an unmet need for acquiring adjuvant therapeutic modalities in pediatric and adolescent populations that will decrease the complications related to impaired glycemic control [9]. Maintaining tight glycemic control in children and adolescents with T1D represents a strenuous task, especially during the period of growth spurt, since insulin antagonizes with growth hormone and glycemic control becomes unmanageable. Strict glycemic control has been shown to reduce the risk of late microvascular complications in T1D. Unfortunately, only a small proportion of individuals reach glycemic targets, and, thus, the need for adjuvant therapeutic modalities is most imperative [10]. The interrelation between the gut microbiome and the glycemic control in apparently healthy populations and in various populations with diabetes is irrevocable [11]. GLP-1, physiologically excreted by the gut epithelium, is in crosstalk with the biochemical messages of the gut microbiome [12]. Gut immune signaling and its afferent and efferent biochemical communications have a great impact on glucose metabolism, insulin concentrations, and glycemic control. Current evidence has led to a significant number of research studies about the impact of probiotics on the glycemic control of pediatric, adolescent, and adult individuals with T1D and other types of the diabetes spectrum [13,14,15,16,17,18].

Type 1 diabetes, as a T-cell-mediated autoimmune disease, entails pro-oxidative, low-grade inflammation even after its diagnosis and treatment [19]. Since the balance between pro- and anti-inflammatory cytokines is impaired, causing inflammation is established and associated with the poor glycemic control observed at the onset of diabetes, during the first year of the disease [20], and most probably later [21]. The function of immune cells, such as dendritic cells (DCs), macrophages, and B and T lymphocytes, seems to be modulated by biochemical “interruptions” of probiotics, playing a definite role in host innate and adaptive immune responses. Theoretically, T regulatory (Treg) cells, which maintain immune homeostasis in the intestinal mucosa, are activated by probiotic-stimulated cytokines. Tregs are potent suppressors of inflammatory immune responses, playing an anti-inflammatory key role in the inflammasome [22]. Therefore, probiotics seem to act as immunomodulating factors and, if administered in appropriate periods and dosages, may ameliorate the progress of diseases or diminish the complications of various disorders, including gastrointestinal (GI) cancers, inflammatory bowel disease (IBD), rheumatoid arthritis, obesity, and diabetes [23].

Probiotics seem to be a promising adjuvant therapy for T1D. Probiotics are biotherapeutics, and their administration delivers active elements to various targets, primarily via the gastrointestinal tract. These elements are active and usually have immunomodulatory and antioxidant effects, possessing antagonistic action against other microorganisms but beneficial effects to the human host. They have many forms, including probiotic-enriched foods. Possible mechanisms of action of probiotics in individuals with T1D include the change in inflammatory dysbiosis to normobiosis, the reduction in pro-oxidative, inflammation-related cytokines, the production of the anti-inflammatory short chain fatty acids (SCFAs), and GLP-1, which protects against insulin resistance. Several studies have evaluated the effects of the administration of probiotics in pediatric and adolescent individuals with T1D, either for prevention [24] or adjuvant treatment [25,26,27,28,29,30], but their results seem to be inconclusive. This systematic review and meta-analysis aimed at shedding light on the effect of the administration of probiotics on the glycemic control of pediatric and adolescent individuals with T1D.

## 2. Materials and Methods

### 2.1. Protocol Registration

The protocol for this systematic review is registered in PROSPERO, an international prospective database for systematic reviews in healthcare. (Registration number: CRD42023451916).

### 2.2. Literature Search Strategy

A literature search was performed from 1 March 2023 until May 2024 in the PubMed, Scopus, and ClinicalTrials.gov databases, without any publication date restrictions. For the PubMed and Scopus searches, *MeSH* terms were used in combinations for either the abstract OR the title. More specifically, the following combinations were employed: “probiotics” *OR* “probiotic agents” *OR* “symbiotic” *OR* “symbiotic agents” *OR* “probiotic bacterium” *AND* “glycemic control” *AND* “glycosylated hemoglobin” *AND* “fasting glucose” *AND* “fasting morning glucose” *AND* “insulin requirements” *AND* “T1D” AND “type 1 diabetes” *AND* “pediatric” *AND* “children” *AND* “adolescents”. The references of full-text articles were screened to limit the possibility of missing pertinent studies. All article abstracts were screened three times repeatedly by two independent reviewers, separately. Papers that met any of the exclusion criteria were excluded, and any controversies between the reviewers were apportioned with agreement during a meeting in which the abstracts were reviewed. All remaining papers were screened again as a full article by the panel of the authors, and conflicts were settled as previously mentioned.

### 2.3. Inclusion Criteria

Randomized controlled trials (RCTs), employing the administration of probiotics in toddlers, children (age range: 2–11 years) and adolescents (age range: 12–19 years) with T1D, written in the English language, providing parameters of glycemic control, such as mean glucose concentrations, irrespectively of the use, or not, of Continuous Glucose Monitoring (CGM) systems and glycosylated hemoglobin, were deemed eligible.

### 2.4. Exclusion Criteria

Review papers, case–control studies, medical hypotheses, letters to the Editor, RCTs with less than 10 individuals per treatment arm, or duplicate papers were excluded.

### 2.5. Quality Assessment of Included Studies

The risk of bias was independently assessed by two authors. Quality measures, assessing the risk of bias, are based on the CASP (Critical Assessment Skills Program) [31] for assessing the quality of RCTs and TRACT checklist (Trustworthiness in Randomized Controlled Trials) for assessing the trustworthiness of the RCTs [32]. Quality assessments were conducted separately by the authors. 

### 2.6. Data Collection Process 

Eligible studies were gathered, and relevant data were extracted from each study in a structured coding scheme using Excel. Data, such as population size, gender and age distribution, mean glucose concentrations and glycosylated hemoglobin (HbA1c) pre- and post-intervention with probiotics, the duration of T1D, the duration of the administration of probiotics, the way of assessment of effectiveness, the side effects associated with the treatment, the dropouts associated with the treatment, and the follow-up period of the individuals, were included, where applicable. A statistician was employed wherever statistical uncertainty or the need for transformation of data emerged, using the appropriate formulas [33]. Regarding data interpretation or usefulness, all authors discussed the study in question to meet agreement.

### 2.7. Data Synthesis

This study is reported according to Preferred Reporting Items for Systematic Reviews and Meta-Analysis (PRISMA) guidelines [34].

### 2.8. Statistical Analysis

The statistical analysis was conducted with SPSS v.29 [35], with permission from the National and Kapodistrian University of Athens, using the default settings of the ‘Meta-analysis’ package. Heterogeneity between studies was assessed using the H^2^ and the *I*^2^ statistics [36]. Data were analyzed using fixed and random effects models. Transformation of median and interquartile range was based on the formula of Wan et al. (2014) [33]. For transformation of standard deviation, the Cochrane Handbook [37] was used.

## 3. Results

### 3.1. Studies Included in the Meta-Analysis

**Figure 1:**
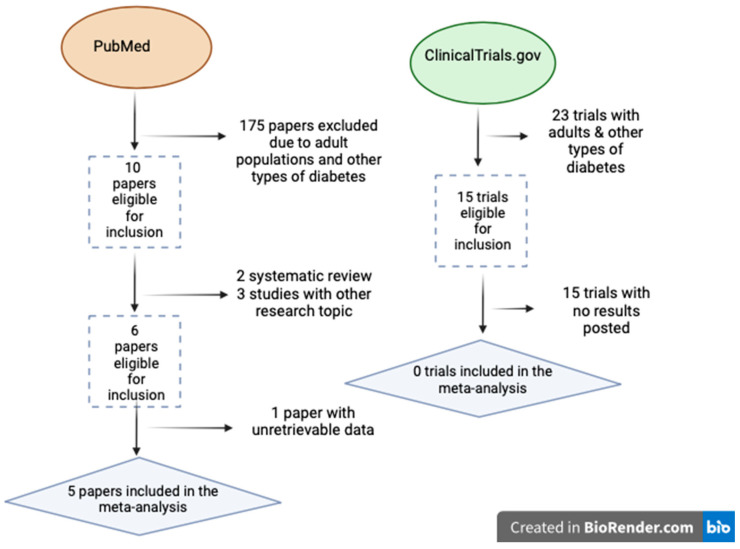
Selection procedure of studies included in the meta-analysis (created with BioRender^®^).

The literature search strategy resulted in 185 papers in the PubMed database and 15 trials in the ClinicalTrials.gov database. After the eligibility assessment, 175 articles were excluded from the PubMed database search as well as all results of the ClinicalTrials.gov database because in the latter, either results were not posted, or they were not completed yet. Altogether, six papers [25,26,27,28,29,30] (Table 1) met the inclusion criteria, but only five papers [25,26,27,29,30] were included in the meta-analysis (Figure 1). Subsequently, this systematic review and meta-analysis included 388 pediatric and adolescent individuals with T1D.

### 3.2. Quality Assessment

The included studies were critically assessed, employing the Critical Appraisal Skills Program (CASP) and the TRACT Screening Checklist, as shown in Table 2 and Table 3.

It seems that the majority of the studies included in this meta-analysis present with adequate but not optimum quality, since they do not report on the adverse effects, and they do not report with detail the exact procedures of the studies. Last but not least, the issue of blindness is omitted.

### 3.3. Main Results

**Figure 2:**
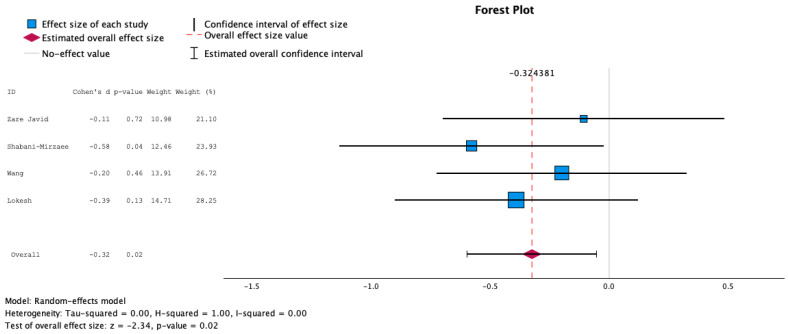
Forest plot presenting the meta-analysis based on standard mean difference (SMD) for the effect of the administration of probiotics on mean glucose concentrations between the studied groups using the random-effects model [25,28,29,30].

**Figure 3:**
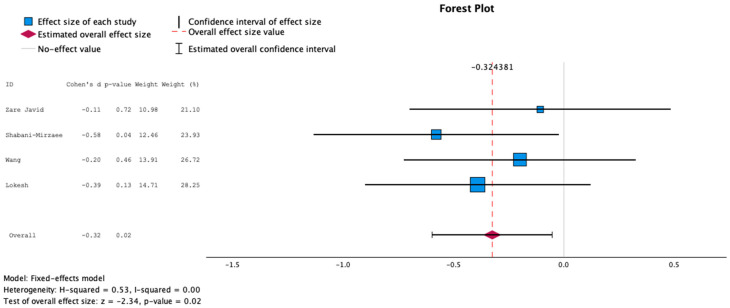
Forest plot presenting the meta-analysis based on standard mean difference (SMD) for the effect of the administration of probiotics on mean glucose concentrations between the studied groups using the fixed-effects model [25,28,29,30].

**Figure 4:**
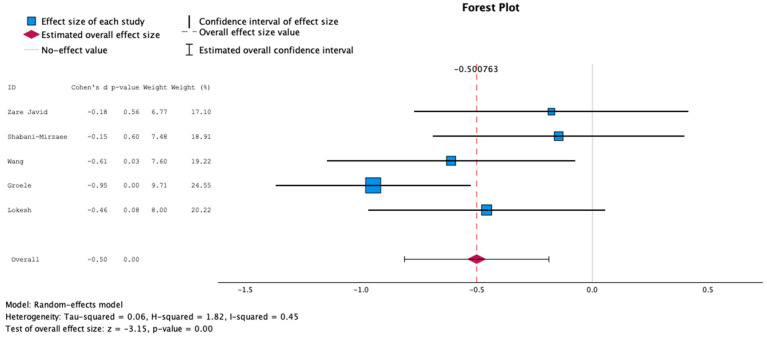
Forest plot presenting the meta-analysis based on standard mean difference (SMD) for the effect of the administration of probiotics on HbA1c concentrations between the studied groups using the random-effects model [25,27,28,29,30].

**Figure 5:**
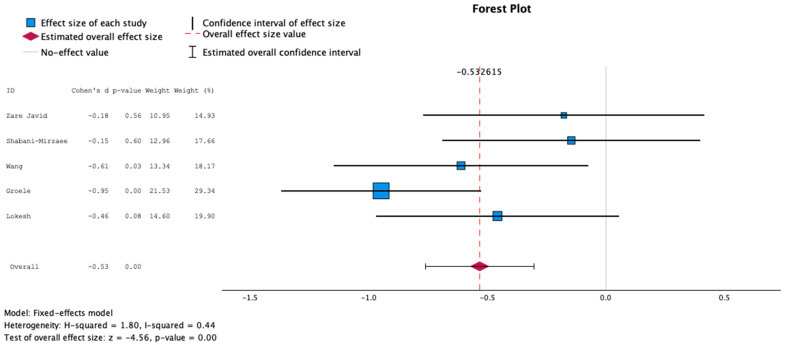
Forest plot presenting the meta-analysis based on standard mean difference (SMD) for the effect of the administration of probiotics on HbA1c concentrations between the studied groups using the fixed-effects model [25,27,28,29,30].

Mean and standard deviation data about glucose concentrations were obtained from four studies [25,28,29,30], as the remaining two studies [26,27] did not provide mean glucose concentrations. Data about mean HbA1c pre- and post-intervention were retrieved from five studies [25,27,28,29,30], whereas one study was not included because of improper data format. The findings about both mean glucose concentrations and HbA1c demonstrated statistically significant negative effect sizes, employing a random and fixed effects model, as shown in Figure 2, Figure 3, Figure 4 and Figure 5, suggesting higher levels of glucose and HbA1c in individuals receiving probiotics than controls.

## 4. Discussion

This systematic review and meta-analysis included RCTs in children and adolescents with T1D, using probiotics as intervention, aiming to explore the effects of probiotics on the glycemic control in terms of mean glucose concentrations and percentage of HbA1c in these individuals. All studies included in the meta-analysis reported mean concentrations of blood glucose and HbA1c, before and after the intervention of the administration of probiotics. In general, the quality and reliability of the studies included in this study were adequate. Models of fixed- and random-effects were employed; however, neither demonstrated statistically significant positive effects on the glycemic control of children and adolescents with T1D. Instead, statistically significant negative effects were revealed, suggesting worse glycemic control in individuals with T1D after the administration of probiotics.

The study by Kumar et al. [26] included pediatric individuals with T1D and demonstrated effectiveness in ameliorating glycemic control. The intervention lasted for 3 months, and follow-up was sustained for a period of 3 months after the termination of the study. Nevertheless, it could not be included in the meta-analysis, as the data about mean glucose concentrations and HbA1c were irretrievable due to the lack of mean values. Also, mean glucose concentrations were mentioned before the intervention but were omitted from the results after the intervention. Instead, the authors assessed the differences in glucose variations and not the mean concentrations of the glucose. In this study [26], the reported adverse effects were minimal and risk-free in a very limited number of individuals, but they failed to report on the compliance of the individuals.

The study of Groele et al. [27] included administration of a multi-strain probiotic in adolescents with newly diagnosed T1D. In this study [27], the administration of probiotics was safe but not effective, neither for the remaining function of beta pancreatic cells nor for the difference in mean HbA1c before and after the intervention [27]. It was included in this meta-analysis since it included the median values of HbA1c, along with Q1 and Q3, before and after the intervention. The appropriate transformations were performed to be included in the meta-analysis. The study of Groele et al. did not include the variables of mean or median glucose concentrations [27]. Groele et al. also included a 6-month follow-up period after the termination of the study, and no adverse effects were reported.

Most of the studies employed multi-strain probiotics, except for the study of Zare Javid et al. [25]. A recent systematic review [38] revealed no significant differences in effectiveness between single-strain or multi-strain probiotics. In addition, it seems that the choice of the appropriate probiotic should be established based on evidence-based trials of efficacy and not on the number of strains or number of colony-forming units (CFUs) of the product. In that systematic review [38], mixtures of multi-strain probiotics were not significantly more effective than single-strain probiotics in most disorders. Almost all the studies, apart from the study of Zare Javid et al. [25], included an intervention period of 24 weeks, which seems like an adequate amount of time to enrich gut microbiota [39], but only one reported the compliance of the individuals without including a follow-up period after the termination of the study. This poses a limitation of this meta-analysis. Other randomized, controlled trials on the administration of probiotics have demonstrated that compliance of the individuals is aligned with the efficacy of the intervention [13].

Wang et al. was the only study employing gut bacteriome analysis and comparing the composition of gut bacteriomes pre- and post-intervention with probiotics. The authors attributed their positive results to the enrichment of the gut microbiome by *Bifidobacterium animalis*, *Lactobacillus salivarius*, and *Akkermansia muciniphila* [28], but they did not comment on the possible mechanism of action of the administered probiotic. This study [28] also included a 3-month follow-up period after the termination of the study. In this follow-up period, the effects of probiotics were sustained. 

The study of Shabani-Mirzaee et al. resulted in mixed results, when the comparison was between the two groups, pre- and post-intervention, but they found statistically significant intra-group differences in the probiotic group before and after the intervention. They administered a multi-strain probiotic supplement for 3 months, but they did not report on the adverse effects, nor did they include a follow-up period [30]. The study of Lokesh et al. resulted in positive effects on delta changes in the glycemic control between the two groups of pediatric individuals with T1D after 6 months with continuous administration of probiotics [29].

The results of this meta-analysis must be interpreted with caution since the quality of the included studies was acceptable, the heterogeneity was moderate, but the number of individuals was limited. Among the included studies, four were assessing the effect size of mean glucose concentrations, and five were assessing the HbA1c. Nevertheless, the number of participants in the included studies was statistically adequate; also, participants did not demonstrate statistically significant differences in their baseline characteristics, thus achieving homogeneity at baseline between the intervention and control groups.

The presence of statistically significant negative effect sizes in the glycemic control in pediatric and adolescent individuals with T1D in the current meta-analysis may be attributable to the inclusion of mixed populations of children and adolescents. Comparing glycemic control between children and adolescents with T1D does include several confounding factors. The progression of puberty denotes the onset of insulin resistance since changes in body composition must be achieved, with fat mass increasing adequately to provide the substrate for sex hormone biosynthesis, contributing to linear growth [40]. In addition, the surges of growth hormone (GH) during puberty represent a great obstacle in managing diabetes at this stage of life because of the antagonistic relationship between the function of the GH and that of the insulin [41]. Adolescence is a critical stage of life in which psychosocial care of diabetes-related compliance is most arduous. In this vicious cycle, environmental cues, such as peer influence and loss of parental control over the behavior, attitude, and diabetes management, come into action, deteriorating compliance and, thus, glycemic control [42,43,44]. Since compliance with the intervention of probiotics was only examined in one study [27], non-compliance may be a major issue for the failure of probiotics in managing glycemic control in adolescents [44].

Another critical issue is the lack and loss of amino acids in children and adolescents with T1D. In 2013, la Marca et al. indicated a carnitine and amino acid deficit may be present before the clinical onset of T1D, probably from birth [45]. Li et al. in 2024 identified 20 gut microbes efficiently depleting amino acids, along with gut microbial metabolic genes that, also, reduce the concentrations of certain amino acids. Due to this gut microbiota-derived depletion, it seems that they affect host amino acid homeostasis, and therefore microbiota genes that deplete amino acid concentrations affect host glucose tolerance via the concentrations of peripheral serotonin [46]. Maybe the specific strain of probiotics used in the included studies of this meta-analysis or the duration of the intervention with probiotics were not suitable for a condition, such as T1D.

Another systematic review about the effect of the administration of probiotics, synbiotics, and prebiotics in pediatric individuals with T1D resulted in inconclusive findings [47]. The authors attributed these ambiguous results to the small number of studies and consequently to the small number of individuals [47]. In contrast with the results of the current meta-analysis, the administration of probiotics and synbiotics in adults with diabetes, either type 1 or type 2 (T2DM), was found to be beneficial. The meta-analysis of Baroni et al. [48] indicated variation in the efficacy of the type of strains used and the studies’ countries of origin. Mixtures of multi-strain probiotics were found to be particularly effective in improving HbA1c concentrations [48]. More specifically, Baroni et al. [48] included studies of adult individuals with either T1D or T2DM, mostly middle-aged with concurrent diabetes-related complications, such as diabetic nephropathy and diabetic foot. In addition, Baroni et al. reported a moderate-to-high heterogeneity of studies, depending on the variable in question [48].

Taylor et al., in a meta-analysis [49], reported no significant reduction in fasting blood glucose (FBG) following probiotic supplementation in females with gestational diabetes (GDM). More specifically, a meta-analysis of four RCTs involving 288 pregnant women with GDM demonstrated that a 6- to 8-week probiotic administration did not improve FBG or low-density lipoprotein cholesterol (LDL-c) concentrations. However, probiotic supplementation in women with GDM was associated with a significant reduction in insulin resistance in terms of HOMA-IR, eventually leading to decreased dosages of any glucose-lowering medication later in their pregnancy [49]. Another systematic review about the effect of the administration of probiotics in prediabetic individuals of different ages, mostly adults, suggested the administration of probiotics as beneficial, since in most studies, healthful effects were demonstrated in the clinical management of individuals with prediabetes and metabolic syndrome.

Different probiotic combinations have also shown beneficial and noticeable effects on glucose homeostasis, lipid profiles, body mass index, and inflammatory markers in subjects with prediabetes and metabolic syndrome, as well as in healthy individuals. Thus, they could be advantageous in either enriching gut microbiota with beneficial microbiota back into normobiosis or contributing to the amelioration of the total biochemical profile of the gut milieu during the prediabetic state [47].

To the best of our knowledge, this is the first meta-analysis about the impact of the administration of probiotics on the glycemic control in pediatric and adolescent individuals with T1D. It has raised questions about the proper methodology of RCTs of probiotics in this special population, while it provides useful insights about the details of the conduct of studies in adolescents with T1D. Adjuvant therapeutic modalities for pediatric and adolescent individuals with T1D should be promptly discovered due to the various unmet health needs of these individuals. To our knowledge, this is the first meta-analysis evaluating the effects of the administration of probiotics on the glycemic control of children and adolescents with T1D. We found statistically significant negative effect sizes of probiotics on the glycemic control of pediatric and adolescent individuals with T1D, but the heterogeneity of the results of the studies does not allow a solid conclusion. Heterogeneity can be attributed to the difference between the physiology of pediatric and adolescent individuals. Notably, no serious adverse effects were reported.

Statistically significant differences should always be compared with actual clinical significance when interventions in individuals with T1D are assessed. Clinical progress in the development of probiotics could be achieved in the future. Better knowledge is needed regarding the active components responsible for each effect, the targets, and the pharmacodynamics and pharmacokinetics of probiotics. Most pharmacokinetic studies have evaluated the survival of probiotic factors in the gastrointestinal tract. Unquestionably, the active components are rarely acknowledged, and their pharmacokinetics, except for those of lactase, cannot be easily assessed.

## 5. Conclusions

Adequately powered studies with extended follow-up periods and employing the proper strains are required for the investigation of the mechanisms of action and the effects of probiotics, particularly concerning diabetes-related complications and metabolic outcomes. Additionally, diligent monitoring and reporting of adverse effects along with compliance are essential for a comprehensive understanding of the role of probiotics separately in pediatric and adolescent individuals with T1D.

## Figures and Tables

**Table 1:** Characteristics of included studies.

Study	N (I/C) *	Age	Probiotic Type	Intervention Period	Dropouts	Meta-Analysis
Zare Javid et al., 2020 [25]	44 (22/22)	4–18 years	Single strain	8 weeks	6	Included
Kumar et al., 2021 [26]	90 (57/49)	2–12 years	Multi-strain	12 weeks	6	Excluded
Groele et al., 2021 [27]	96 (48/48)	8–17 years	Multi-strain	24 weeks	9	Included
Wang et al., 2022 [28]	56 (27/29)	6–18 years	Multi-strain	24 weeks	3	Included
Shabani-Mirzaee et al., 2023 [30]	52 (26/26)	2–16 years	Multi-strain	12 weeks	0	Included
Lokesh et al., 2024 [29]	50 (27/23)	2–12 years	Multi-strain	24 weeks	10	Included

* I = intervention group; C = control group.

**Table 2:** Quality report of the Critical Appraisal Skills Program (CASP) of the included studies.

CASP	Zare Javid et al., 2020 [25]	Kumar et al., 2021 [26]	Groele et al., 2021 [27]	Wang et al., 2021 [28]	Shabani-Mirzaee et al., 2023 [30]	Lokesh et al., 2024 [29]
1. Did the study address a clearly focused research question?	Yes	Yes	Yes	Yes	Yes	Yes
2. Was the assignment of participants to interventions randomized?	Yes	Yes	Yes	Yes	Yes	Yes
3. Were all participants who entered the study accounted for at its conclusion?	No	Yes	No	Yes	Yes	Yes
4. Were the participants ‘blind’ to the intervention they were given?	Cannot tell	Yes	Yes	Yes	Yes	Yes
Were the investigators ‘blind’ to the intervention they were giving to participants?	Cannot tell	Yes	Cannot tell	Cannot tell	Yes	Yes
Were the people assessing/analyzing outcomes ‘blinded’?	Cannot tell	Cannot tell	Cannot tell	Cannot tell	Yes	Yes
5. Were the study groups similar at the start of the randomized controlled trial?	No	Yes	Yes	Yes	Yes	Yes
6. Apart from the experimental intervention, did each group receive the same level of care (that is, were they treated equally)?	Yes	Yes	Cannot tell	Cannot tell	Yes	Cannot tell
7. Were the effects of the intervention reported comprehensively?	Yes	No	Yes	Yes	Yes	Yes
8. Was precision of the estimate of the intervention’s treatment effect reported?	Yes	No	No	No	No	No
9. Do the benefits of the experimental intervention outweigh the harms and costs?	Cannot tell	Cannot tell	Cannot tell	Cannot tell	Cannot tell	Cannot tell
10. Can the results be applied to your local population/in your context?	Yes	Yes	Cannot tell	Cannot tell	Cannot tell	Cannot tell
11. Would the experimental intervention provide greater value to the people in your care than any of the existing interventions?	Cannot tell	Cannot tell	Cannot tell	Cannot tell	Cannot tell	Cannot tell

**Table 3:** Quality report of the trustworthiness of the included studies according to the TRACT screening checklist.

Study	Gover Nance	Author Group	Plausibility of Intervention Usage	Time Frame	Drop-Out Rates	Baseline Characteristics	Outcomes
Zare Javid et al., 2020 [25]	No concerns	Some concerns	No concerns	Major concerns	No concerns	No concerns	Major concerns
	Some concerns	No concerns	Major concerns	Major concerns	Major concerns	No concerns	No concerns
	No concerns	No concerns				No concerns	No concerns
						No concerns	
Kumar et al., 2021 [26]	No concerns	Some concerns	No concerns	Major concerns	No concerns	No concerns	No concerns
	No concerns	No concerns	No concerns	No concerns	No concerns	No concerns	No concerns
	No concerns	No concerns				No concerns	No concerns
						No concerns	
Groele et al., 2021 [27]	No concerns	Some concerns	No concerns	No concerns	Major concerns	No concerns	No concerns
	Some concerns	No concerns	No concerns	No concerns	Major concerns	No concerns	No concerns
	No concerns	No concerns				No concerns	No concerns
						No concerns	
Wang et al., 2021 [28]	No concerns	No concerns	No concerns	Major concerns	No concerns	Major concerns	Major concerns
	No concerns	No concerns	Major concerns	Major concerns	No concerns	No concerns	
	No concerns	No concerns				No concerns	No concerns
						Major concerns	No concerns
Shabani-Mirzaee et al., 2023 [30]	No concerns	No concerns	No concerns	Some concerns	Major concerns	Some concerns	No concerns
	Some concerns	No concerns	Major concerns	No concerns	No concerns	Some concerns	Some concerns
	No concerns	No concerns				Some concerns	No concerns
						No concerns	
Lokesh et al., 2024 [29]	No concerns	No concerns	No concerns	No concerns	No concerns	No concerns	No concerns
	No concerns	No concerns	Major concerns	No concerns	No concerns	No concerns	No concerns
	Some concerns	No concerns				No concerns	No concerns
						No concerns	
